# Supplementary material for: Phenotypic analysis of the unstimulated in vivo HIV CD4 T cell reservoir
Source: eLife. 2020 Sep 29;9:e60933. doi: 10.7554/eLife.60933 (PMC7524554; doi:10.7554/eLife.60933)
Supplement: Supplementary file 1. — Antibodies were either purchased from the indicated vendor or prepared in-house using commercially available MaxPAR conjugation kits per manufacturer’s instructions (Fluidigm). [file elife-60933-supp1.docx]

**Supplementary File 1:** **List of** **CyTOF antibodies used in study**

| **Antigen Target** | **Clone** | **Elemental Isotope** | **Vendor** |
| --- | --- | --- | --- |
| HLADR | TÜ36 | Qdot (112Cd) | Life Technologies |
| RORγt* | AFKJS-9 | 115 In | In-house |
| CD49d (α4) | 9F10 | 141Pr | Fluidigm |
| CTLA4* | 14D3 | 142Nd | In-house |
| NFAT* | D43B1 | 143Nd | Fluidigm |
| CCR5 | NP6G4 | 144Nd | Fluidigm |
| BIRC5* | 91630 | 145Nd | In-house |
| SAMHD1* | Ag3287 Immunogen | 145Nd | In-house |
| Ki67* | B56 | 146Nd | In-house |
| CD95 | BX2 | 146Nd | In-house |
| BCL6* | K112-91 | 146Nd | In-house |
| CD7 | CD76B7 | 147Sm | Fluidigm |
| ICOS | C398.4A | 148Nd | Fluidigm |
| Tbet* | 4B10 | 149Sm | In-house |
| Gag (mixture)* | 71-31, 91-5, 241-D, AG3.0 | 150Nd | In-house |
| CD2 | TS1/8 | 151Eu | Fluidigm |
| CD103 | Ber-ACT8 | 151Eu | Fluidigm |
| Gag (28B7)* | 28B7 | 152Sm | In-house |
| CD62L | DREG56 | 153Eu | Fluidigm |
| TIGIT | MBSA43 | 154Sm | Fluidigm |
| CCR6 | 11A9 | 155Gd | In-house |
| Gag (KC57)* | FH190-1-1 | 156 Gd | In-house |
| CD8 | RPA-T8 | 157Gd | In-house |
| CD19 | HIB19 | 157Gd | In-house |
| CD14 | M5E2 | 157Gd | In-house |
| OX40 | ACT35 | 158Gd | Fluidigm |
| CCR7 | G043H7 | 159Tb | Fluidigm |
| CD28 | CD28.2 | 160Gd | Fluidigm |
| CD45RO | UCHL1 | 161Dy | In-house |
| CD69 | FN50 | 162Dy | Fluidigm |
| CRTH2 | BM16 | 163Dy | Fluidigm |
| PD-1 | EH12.1 | 164Dy | In-house |
| CD127 | A019D5 | 165Ho | Fluidigm |
| CXCR5 | RF8B2 | 166Er | In-house |
| CD27 | L128 | 167Er | Fluidigm |
| CD30 | BERH8 | 168Er | In-house |
| CD45RA | HI100 | 169Tm | Fluidigm |
| CD3 | UCHT1 | 170Er | Fluidigm |
| CD57 | HNK-1 | 171Yb | In-house |
| CD38 | HIT2 | 172Yb | Fluidigm |
| α4β7 | Act1 | 173Yb | In-house |
| CD4 | SK3 | 174Yb | Fluidigm |
| CXCR4 | 12G5 | 175Lu | Fluidigm |
| CD25 | M-A251 | 176Yb | In-house |
| Gag (KC57)* | FH190-1-1 | 209 Bi | In-house |

*Intracellular antibodies

Antibodies were either purchased from the indicated vendor or prepared in-house using commercially available MaxPAR conjugation kits per manufacturer’s instructions (Fluidigm).
