# Supplementary material for: Phenotypic analysis of the unstimulated in vivo HIV CD4 T cell reservoir
Source: eLife. 2020 Sep 29;9:e60933. doi: 10.7554/eLife.60933 (PMC7524554; doi:10.7554/eLife.60933)
Supplement: Supplementary file 2. — Table of participant characteristics listing gender, ethnicity, age, year of first HIV+ test, viral load, CD4 count at the time of sampling, ART regimen, and specimen type used in this study. [file elife-60933-supp2.docx]

| Patient ID | Gender | Ethnicity | Age | Year of first HIV+ test | Viral Load  (copies/ml) | CD4 count (per/mm^3^) | ART regimen | Specimen type |
| --- | --- | --- | --- | --- | --- | --- | --- | --- |
| PID1695* | Female | Mixed | 40 | 2010 | <40 | 710 | FTC/TDF, ATV, RTV | Leukapheresis |
| PID3010* | Male | AA | 54 | 1990 | <40 | 902 | DRV, RTV, RPV/TAF/FTC | Leukapheresis  FNA |
| PID5003* | Male | Hispanic | 48 | 1993 | <40 | 289 | ATV, ABC/TCV/3TC | Leukapheresis |
| PID2053* | Male | Asian | 60 | 1991 | <40 | 333 | FTC/TAF, TCV | Leukapheresis |
| PID01223 | Female | AA | 39 | 2000 | <20 | 989 | BIC/FTC/TAF | Gut |
| PID1128 | Male | AA | 69 | 1996 | <40 | 318 | BIC/FTC/TAF | Gut |
| PID2511 | Male | White | 51 | 2001 | <40 | 330 | TCV/RPV | Gut |
| PID2402 | Male | AA | 43 | 2004 | <40 | 545 | BIC/FTC/TAF | Leukapheresis  Gut |
| PID2161 | Male | White | 71 | 1985 | <40 | 677 | 3TC/DRV/RTV/TCV | Leukapheresis |
| PID2375 | Male | White | 71 | 1989 | <40 | 594 | EFV/TDF/FTC | Leukapheresis |
| PID2781 | Male | White | 43 | 2009 | <40 | 518 | ABC/TCV/3TC | Leukapheresis |

**Supplementary File 2: Participant characteristics**

*Complete CyTOF/PP-SLIDE analyses + validations for these leukapheresis specimens

Table of participant characteristics listing gender, ethnicity, age, year of first HIV+ test, viral load, CD4 count at time of sampling, ART regimen, and specimen type used in this study.
