# Supplementary material for: Phenotypic analysis of the unstimulated in vivo HIV CD4 T cell reservoir
Source: eLife. 2020 Sep 29;9:e60933. doi: 10.7554/eLife.60933 (PMC7524554; doi:10.7554/eLife.60933)
Supplement: Transparent reporting form [file elife-60933-transrepform.docx]

***eLife’s* transparent reporting form**

We encourage authors to provide detailed information *within their submission* to facilitate the interpretation and replication of experiments. Authors can upload supporting documentation to indicate the use of appropriate reporting guidelines for health-related research (see [EQUATOR Network](http://www.equator-network.org/%20)), life science research (see the [BioSharing Information Resource](https://biosharing.org/)), or the [ARRIVE guidelines](http://www.plosbiology.org/article/info:doi/10.1371/journal.pbio.1000412) for reporting work involving animal research. Where applicable, authors should refer to any relevant reporting standards documents in this form.

If you have any questions, please consult our Journal Policies and/or contact us: [editorial@elifesciences.org](mailto:editorial@elifesciences.org).

**Sample-size estimation**

- You should state whether an appropriate sample size was computed when the study was being designed
- You should state the statistical method of sample size computation and any required assumptions
- If no explicit power analysis was used, you should describe how you decided what sample (replicate) size (number) to use

Please outline where this information can be found within the submission (e.g., sections or figure legends), or explain why this information doesn’t apply to your submission:

No explicit power analysis was used, as sample size calculations are difficult for CyTOF-based studies because high-dimensional CyTOF datasets harness parametric dimensionality to gain statistical power. Previously, data from 3-5 donors gave sufficient statistical power for high-dimensional analysis in published CyTOF datasets that had similar numbers of parameters (Wong et al, Cell Rep 11:1822; Sen et al Cell Reports 8:633; Cavrois et al Cell Reports 20:984). In our study, the we conducted CyTOF analyses on a total of 10 donors, four of which were used to validate the CyTOF results through sorting experiments. Furthermore, the predictions made from the CyTOF data were validated on 4 additional untested donors not analyzed by CyTOF, to demonstrate the generalizability of our conclusions.

**Replicates**

- You should report how often each experiment was performed
- You should include a definition of biological versus technical replication
- The data obtained should be provided and sufficient information should be provided to indicate the number of independent biological and/or technical replicates
- If you encountered any outliers, you should describe how these were handled
- Criteria for exclusion/inclusion of data should be clearly stated
- High-throughput sequence data should be uploaded before submission, with a private link for reviewers provided (these are available from both GEO and ArrayExpress)

Please outline where this information can be found within the submission (e.g., sections or figure legends), or explain why this information doesn’t apply to your submission:

All figures show data from each donor that was tested; in no case did we only show a single “representative” donor. All error bars and statistical analyses corresponded to biological (not technical) replicate samples that originated from different donors. The kind of statistical test used for the figures is detailed in the figure legend and much more extensively in the methods section. No outliers were encountered. No data were excluded. The raw data used in the paper are available through the following link: https://datadryad.org/stash/share/V6g8JRc2JvGtTqFRJi3fs6vE8lsef5eNJalDdbHW61g

**Statistical reporting**

- Statistical analysis methods should be described and justified
- Raw data should be presented in figures whenever informative to do so (typically when N per group is less than 10)
- For each experiment, you should identify the statistical tests used, exact values of N, definitions of center, methods of multiple test correction, and dispersion and precision measures (e.g., mean, median, SD, SEM, confidence intervals; and, for the major substantive results, a measure of effect size (e.g., Pearson's r, Cohen's d)
- Report exact p-values wherever possible alongside the summary statistics and 95% confidence intervals. These should be reported for all key questions and not only when the p-value is less than 0.05.

Please outline where this information can be found within the submission (e.g., sections or figure legends), or explain why this information doesn’t apply to your submission:

The exact statistical analyses used and their justifications are presented within the Figure Legends and Methods. We always presented raw and not normalized data. P-values were reported as appropriate.

(For large datasets, or papers with a very large number of statistical tests, you may upload a single table file with tests, Ns, etc., with reference to sections in the manuscript.)

**Group allocation**

- Indicate how samples were allocated into experimental groups (in the case of clinical studies, please specify allocation to treatment method); if randomization was used, please also state if restricted randomization was applied
- Indicate if masking was used during group allocation, data collection and/or data analysis

Please outline where this information can be found within the submission (e.g., sections or figure legends), or explain why this information doesn’t apply to your submission:

Within each experiment, internal controls were always implemented. For example, for each donor sample, we always included unstimulated “atlas” cells from that exact same donor and those cells were processed in parallel with samples that were stimulated ex vivo. In no case did we compare atlas vs. reactivated cells from different donors or tissues.

**Additional data files (“source data”)**

- We encourage you to upload relevant additional data files, such as numerical data that are represented as a graph in a figure, or as a summary table
- Where provided, these should be in the most useful format, and they can be uploaded as “Source data” files linked to a main figure or table
- Include model definition files including the full list of parameters used
- Include code used for data analysis (e.g., R, MatLab)
- Avoid stating that data files are “available upon request”

Please indicate the figures or tables for which source data files have been provided:

We have made the raw CyTOF datasets generated from this study publicly available through the public repository Dryad. They can be accessed via the following link: https://datadryad.org/stash/share/V6g8JRc2JvGtTqFRJi3fs6vE8lsef5eNJalDdbHW61g
